# Non-melanoma skin cancer among ethnic German immigrants (resettler) from the former Soviet Union: a cohort study from 1990 to 2007

**DOI:** 10.1186/s13690-022-00842-1

**Published:** 2022-03-18

**Authors:** Evgenia Markeeva-Ilisevic, Bernd Holleczek, Heiko Becher, Volker Winkler

**Affiliations:** 1 Medical Faculty of Heidelberg, Heidelberg, Germany; 2 Saarland cancer registry, Saarbrücken, Germany; 3 Institute of Medical Biometry and Epidemiology, The University Medical Center Hamburg-Eppendorf, Hamburg, Germany; 4 Heidelberg Institute of Global Health, Heidelberg, Germany

**Keywords:** Non-melanoma skin cancer, Basal cell carcinoma, Squamous cell carcinoma, Resettlers, UV radiation

## Abstract

**Background:**

UV radiation is a significant risk factor for non-melanoma skin cancer (NMSC). Ethnic Germans (resettlers) who immigrated to Germany from the former Soviet Union may have had a relatively high UV light exposure and thus a higher risk of developing NMSC. We compared the incidence of NMSC in a resettler cohort with the general population of the *Saarland* (Federal state of Germany) in relation to tumour location.

**Methods:**

All new NMSC cases (resettler cohort and total population) between 1990 and 2007 were retrieved from the *Saarland* cancer registry and classified according to sex, histology, and location. The classification used for tumour location approximated the previous UV exposure. Age-standardized incidence rates (ASR) for the general population and standardized incidence ratios (SIR) for resettlers compared to the general population were calculated and modelled using Poisson regression.

**Results:**

Sex-specific overall SIR indicated a significant increase in female resettlers (SIR 1.31 (95% CI 1.02–1.67)) which can mostly be attributed to an increased incidence of squamous cell carcinoma. The regression analysis showed that among resettlers the risk of developing tumours in UV-exposed skin areas was 2.16 (95% CI 1.35–3.45) higher compared to the general population.

**Conclusions:**

Female resettlers have a higher risk to be diagnosed with NMSC than the general German population. Based on the observed distribution of tumour location, it is suspected that UV exposure contributed significantly to this risk.

## Background

Non-melanoma skin cancer (NMSC) is the most common type of cancer in humans. However, the term NMSC is not clearly defined. It represents a group of cutaneous malignancies including basal cell carcinomas (BCC), cutaneous squamous cell carcinomas (SCC) and some other types (inter alia extramammary Paget's disease, Merkel cell carcinoma, skin adnexal carcinomas). In Germany, about 230,000 people developed NMSC in  2016 [1]. BCC is a slowly destructive and infiltrative tumour that metastasises very rarely. With an incidence of approximately 200 new cases per 100,000 people in 2013, it was the most common skin cancer entity in Germany [2]. SCC represent the second most common non-melanoma skin cancer showing an indolent clinical course. This type of skin neoplasia begins typically with a premalignant lesion – actinic keratosis. Metastases of SCC were observed in a prospective study with a frequency of 4% within 43 months [3]. Tumours other than BCC/SCC represent a small inhomogeneous group of skin neoplasms with different and not always clear aetiology.

The most important risk factor for the development of BCC and SCC is ultraviolet (UV) radiation exposure. UV radiation is considered an obligate carcinogen for humans [4]. In the case of BCC, it is assumed that intermittent irradiation, especially in childhood, but also intensive tanning in the context of leisure activities is decisive [5–8]. In the case of SCC, the cumulative dose over a lifetime is of particular importance [8]. The meaning of UV exposure for the tumours other than BCC/SCC depends on entity and is presumably of subordinate importance.

According to the Federal Statistical Office, approximately 25.5% of the German population has a migration background [9]. Of these, the so-called *Aussiedler* (resettlers) with their own migration experience represent the second largest group (approx. 2.6% of the total German population) [10]. Resettlers are immigrants of German descent who came from states of the former Eastern Bloc. Until 1989 they mostly came from Poland and Romania, however their majority immigrated from the states of the former Soviet Union after 1990, mainly from Kazakhstan and the Russian Federation [11]. In previous studies, it has been shown, that resettler have higher risk in comparison to the general German population to develop stomach cancer and lung cancer but a lowered risk for breast cancer [12, 13].

NMSC is the most common form of cancer and is of particular importance for public health. In this study, we investigated the incidence of NMSC in ethnic Germans from the former Soviet Union in comparison to the general German population. Of specific interest are UV-exposed tumours, as there could be an increased risk among resettlers due to a higher UV exposure in their countries of origin.

## Methods

Within the framework of a retrospective register-based study, we investigated the occurrence of NMSC in a cohort of resettlers in comparison to the total population of the *Saarland* (Federal state of Germany). The cohort consists of 18,619 resettlers (approximately 64% all resettlers) who immigrated to the Saarland between the years 1990 and 2005 from countries of the former Soviet Union. The dataset was directly obtained from all local reception centres and contained name, date of birth, issue date of a German passport as an approximation for date of migration, sex, country of birth and first city of residence [14].

The observation period started with the date of migration, which was determined by the date of issue of the German passport [12, 15]. To avoid bias due to the introduction of skin cancer screening in Germany, December 31, 2007, was defined as the end of follow-up for the present study.

The regional registration authorities were asked about the vital status and out-migration from the study area (Saarland), which were considered to be the end of follow-up for the study.

Data on skin cancer incidence (every first NMSC-case per person) were provided by the Saarland cancer registry. The identification of the resettler cohort among the Saarland population was done with the help of a computer-assisted record linkage procedure by the comparison of name components and their phonetic codes as well as information on sex, date of birth, and place of residence [14]. All cases of skin cancer of the total *Saarland* population including resettler cohort were coded according to the International Statistical Classification of Diseases and Related Health Problems 9^th^ Revision (ICD-9) and the morphological code of the International Classification of Disease for Oncology 2^nd^ Revision (ICD-O 2). Histological types were coded as BCC, SCC and Other using the ICD-O 2 classification. UV light exposure was approximated by tumour location using the ICD-9 classification (see Table 1) [6, 16].

**Table 1:** Classification of skin tumours by histological type and tumour location according to ICD-O 2 and ICD-9

Group	Classification
**Histological type (ICD-O2)**	
basal cell carcinoma (BCC)	8090/3; 8091/3; 8092/3; 8093/3; 8094/3; 8095/3
squamous cell carcinoma(SCC)	8070/2; 8070/3; 8071/3; 8072/3; 8074/3; 8075/3; 8076/3; 8051/3; 8052/3
Other	Non-specific diagnoses: 8000/1; 8000/3; 8010/3; 8230/3 Other diagnoses: 8021/3; 8147/3; 8260/3; 8490/3; 8562/3; 8390/3; 8400/3; 8402/3; 8403/3; 8407/3; 8410/3; 8420/3; 8480/3; 8560/3; 8890/3; 8102/3; 8542/3; 8810/3; 8830/3; 8940/3; 9140/3; 8190/3; 8247/3; 8832/3; 9080/3; 8200/3
**Approximated UV light exposure** **(Tumour location (ICD-9))**	
UV-exposed	173.0; 173.1; 173.2; 173.3; 173.4 (Lip, eyelid including canthus, ear and external auditory canal, oth. and unspec. parts of face, scalp and skin of neck)
UV-unexposed	173.5; 173.6; 173.7; 173.8 (Trunk except scrotum, upper limb, including shoulder, lower limb, including hip, oth. spec. sites of skin)
Unknown	173.9 (unspecified)

Person-years of time under risk for the resettler cohort were calculated separately for both sexes, calendar years and 5-year age groups.

Age-standardised incidence rates (ASR) of the Saarland population were calculated based on the mid-year population and the European Standard Population [17] for the following categories, separately by sex and calendar year: all skin cancers, histological type (BCC, SCC, Other), approximated UV light exposure (UV-exposed, UV-unexposed, Unknown). Standardised incidence ratios (SIR) were calculated for resettlers in comparison to the Saarland population for the same categories as well as by sex and two time periods (1990–1998, 1999–2007). For this purpose, the number of skin cancer cases observed among the resettlers was divided by the expected number, which results from the age- and calendar year-specific Saarland rates and the person-years of the cohort. Exact 95% confidence intervals (95% CI) were calculated for ASR and SIR [18, 19].

Finally, the SIR was modelled using Poisson regression to examine independent effects. The observed number of diagnoses served as the dependent variable, the expected number as the offset and standard errors were scaled using Pearson's chi-square. Independent variables were calendar year (continuous as year minus 1990), sex, histological type, and approximated UV radiation exposure. All statistical analyses were performed using Stata 15.1 IC (Copyright 1985–2017 StataCorp LLC, StataCorp, 4905 Lakeway Drive, College Station, Texas 77,845 USA).

## Results

The cohort of resettlers consists of 18,619 persons, of whom 8,976 were men and 9,643 women. During the observation period it accumulated 177,644.2 person-years with a mean observation time of 9.5 years. A total of 18,575 new cases of skin cancer were identified in the Saarland during the same period, of which 51.5% were in men. Among resettlers, 43.3% of the 120 diagnoses were among men (see Table 2). The mean age at diagnosis was 69 years in the general population and 68 years in resettlers. Overall, 80.5% of all skin cancer diagnoses were BCC and 17.4% SCC. Among resettlers, it was 75.8% and 23,3%, respectively. The approximated UV exposure status (tumour location) showed an unequal distribution between resettlers and the general population. Rather UV-exposed tumours represented 66.7% of the diagnoses in the Saarland population (22.9% unexposed, 10.4% unknown) and 76.7% (14.2% unexposed, 9.2% unknown) in resettlers.

**Table 2:** Incident skin cancer in the resettler cohort and in the general Saarland population for the observation period 1990 to 2007

	**Men**		**Women**		**Total**	
	**Saarland**	**Resettlers**	**Saarland**	**Resettlers**	**Saarland**	**Resettlers**
Skin cancer diagnoses [N]	9,564	52	9,011	68	18,575	120
Mean age at diagnoses (median, range)	68 (69, 20–102)	66 (68.5, 39–85)	70 (72, 16–105)	69 (72, 37–89)	69 (71, 16–105)	68 (70, 39–88)
Mid-year population	518,712.6		551,080.9		1,069,793.5	
Person-years		85,801.3		91,842.9		177,644.2
**Histological type [N (%)]**						
basal cell carcinoma (BCC)	7,581 (79.3)	39 (75)	7,381 (81.9)	52 (76.5)	14,962 (80.5)	91 (75.8)
squamous cell carcinoma (SCC)	1,810 (18.9)	12 (23.1)	1,427 (15.8)	16 (23.5)	3,237 (17.4)	28 (23.3)
Other	173 (1.8)	1 (1.9)	203 (2.3)	0	376 (2.02)	1 (0.8)
**Approximated UV exposure (tumour location) [N (%)]**						
UV-exposed	6,342 (66.3)	38 (73.1)	6,049 (67.1)	54 (79.4)	12,391 (66.7)	92 (76.7)
UV-unexposed	2,237 (23.4)	10 (19.2)	2,009 (22.3)	7 (10.3)	4,246 (22.9)	17 (14.2)
Unknown	985 (10.3)	4 (7.7)	953 (10.6)	7 (10.3)	1,938 (10.4)	11 (9.2)

**Fig. 1:**
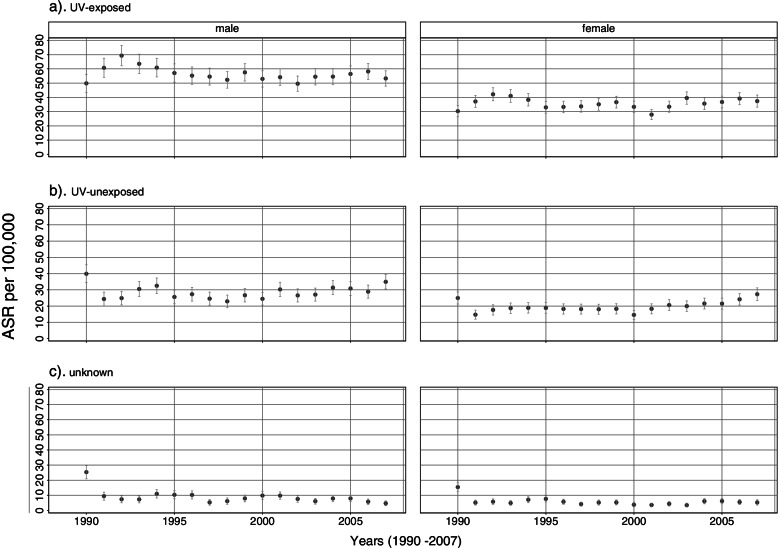
Yearly age-standardized incidence rates (European standard) (ASR) of non-melanoma skin cancer with 95% confidence intervals among the general population of the Saarland separated by the approximated UV exposure (tumour location) and sex for the observation period 1990 to 2007

Figure 1 shows the temporal development of ASR of Saarland population according to approximated UV exposure (tumour location) and sex for the general population of the Saarland. The ASR per 100,000 persons is relatively stable over the observation period (year 2007) with 58.6 (95% CI 57.2–59.9) for men and 37.1 (95% CI 36.1–38.0) for women for UV-exposed locations. For comparison, ASRs of UV-exposed locations among resettlers (estimated by means of SIR) were 61.4 (95% CI 40.6–82.2) for men and 57.9 (95% CI 42.3–73.5) for women.

For total NMSC, the standardised incidence ratios (SIR) comparing resettlers to the general population were not increased (see Table 3), but when looking sex-specifically, there was an increase for female resettlers (1.31 (95% CI 1.02–1.67)), especially in the first period (1990–1998). When separated according to the approximated UV exposure status, there was again a higher SIR for women. When separated by histological type, higher SIR were also found for SCC among both sexes together and among women.

**Table 3:** Standardised incidence ratios (SIR) with 95% confidence intervals of non-melanoma skin cancers for resettlers compared to the general population of Saarland by approximated UV exposure (tumour location), histological type, sex, and two time periods

	**Men**		**Women**		**Total**	
	N	SIR (95% CI)	N	SIR (95% CI)	N	SIR (95% CI)
**Total**	52	0.97 (0.72–1.27)	68	1.31 (1.02–1.67)	120	1.14 (0.94–1.36)
**Time period**						
1990–1998	19	1.03 (0.62–1.61)	30	1.70 (1.15–2.43)	49	1.36 (1.01–1.80)
1999–2007	33	0.93 (0.64–1.32)	38	1.11 (0.79–1.53)	71	1.02 (0.80–1.29)
**Histological type**						
basal cell carcinoma (BCC)	39	0.90 (0.64–1.22)	52	1.17 (0.87–1.53)	91	1.03 (0.83–1.27)
squamous cell carcinoma (SCC)	12	1.29 (0.66–2.25)	16	2.51 (1.44–1.08)	28	1.78 (1.19–2.58)
Other	1	1.34 (0.03–7.44)	0	0 (0–4.64)	1	0.65 (0.02–3.62)
**Approximated UV exposure (tumour location)**						
UV-exposed	38	1.09 (0.77–1.49)	54	1.65 (1.24–2.15)	92	1.36 (1.09–1.70)
UV-unexposed	10	0.73 (0.35–1.30)	7	0.49 (0.20–1.02)	17	0.61 (0.35–0.97)
Unknown	4	0.81 (0.23–2.07)	7	1.45 (0.58–2.99)	11	1.13 (0.56–2.00)

Table 4 presents the independent variables influencing the SIR modelled by Poisson regression. All independent variables show a significant (*p* < 0.05) association with the SIR. On UV-exposed skin areas, the SIR was independently increased by a factor of 2.16 (95% CI 1.35–3.45) compared to non-exposed skin areas. Furthermore, it should be emphasised that the SIR decreases every year.

**Table 4:** Poisson regression results for modelling the standardised incidence ratios for resettlers compared to the general population of Saarland

	**RRR**^a^	**95% CI**	***p*-value**
**Calendar year**			
(Year – 1990)	0.96	0.92–0.99	0.014
**Sex**			
men	1		0.032
women	1.43	1.03–1.99	
**Histological type**			
Basal cell carcinoma	1		0.012
Squamous cell carcinoma	1.77	1.2–2.61	
Other	0.66	0.11–3.93	
**Approximated UV exposure (tumour location)**			
UV-unexposed	1		0.006
UV-exposed	2.16	1.35–3.46	
Unknown	1.74	0.88–3.48	
**Constant (SIR at baseline)**	0.75	0.41–1.39	0.365

^a^*RRR* Relative Risk Ratio

Baseline: men, basal cell carcinoma, UV-unexposed, year 1990

## Discussion

In the present study, we investigated the incidence of non-melanoma skin cancer among resettlers from the former Soviet Union with a special focus on tumour location and previous UV exposure. It is shown that skin cancer developed more frequently in UV-exposed skin areas of female resettlers compared to the female general population. Accordingly, there was also a higher proportion of squamous cell carcinomas. Over time skin cancer incidence decreased among resettlers while rates of the general population remained largely stable.

Factors that significantly determine individual UV exposure are skin type, geographical location of residence, leisure time behaviour and occupational exposure. With regard to skin type, there are no known differences between resettlers and Germans; both are assigned to the Caucasian skin type [20].

Historically, resettlers immigrated mainly from Kazakhstan and the Russian Federation, and there mainly from Siberia. In Kazakhstan, the climate is strictly continental and the UV index in Astana (now Nur-Sultan, the capital of Kazakhstan) is 6–7 (high) during June and July [21, 22]. However, Astana is located more in the northern part of Kazakhstan. Cities in southern Kazakhstan, such as Almaty (former capital of the Kazakh Soviet Socialist Republic) show a UV index in July between 8–10 (very high) up to 11 (extreme) [22]. In addition to time of year and day, the UV index depends mainly on latitude and is directly associated with the risk of NMSC [23–25]. The most frequent tumour localisations of BCC and SCC were reported in the head and neck region among Astana residents [20]. As for climatic conditions in Russia, it is not possible to generalise due to the size of the country. One of the most important settlement regions of ethnic Germans in Russia are areas around the city of Omsk and the Altai region in Siberia, which are located around 54° and 60° north latitude, respectively. There, a UV index of max. 7 (high) is reached [26]. By comparison, the average value for the UV index in July 2019 in the city of *Wiesbaden* (the closest measuring station with a documented UV index history for the *Saarland*) was 6 (high) [27].

In the former Soviet Union, public life usually took place outdoors in the summer with limited leisure activities and crowded living conditions. In addition, work in one's own garden was common as an important place to grow food due to the lack of goods in stores. Furthermore, in Soviet times, forced labour in fields was also part of everyday life in the Asian republics for schoolchildren, students, and state employees [28, 29]. The work usually took place at harvest time in the fall over a period of several weeks. Those affected at times lived in tent camps and worked outside every day, regardless of weather conditions. Outdoor sports activities probably played a subordinate role in this cohort. In Germany, 67% of resettlers did not engage in regular physical activity according to a survey [30]. In general, we lack reliable information regarding NMSC incidence in the Union of Soviet Socialist Republics (USSR). Although cancer registration is available since seventies [31] most data is not accessible and not available for ethnic Germans.

Slightly more than half of the ethnic German immigrants completed vocational training in their countries of origin in the agricultural sector or in other skilled trades, which are often practiced outdoors. On the other hand, a relatively large number of ethnic German women have higher educational qualifications, which are presumably more likely to be practiced indoors (29% versus 30% of women without a migrant background) [11]. It can be noted that a European style of dress was also propagated and maintained in Islamic regions such as Kazakhstan [32]. Furthermore, it can be assumed that the dangers of excessive UV exposure were largely unknown in the Soviet population and thus preventive measures were neglected [33, 34].

For non-UV-exposed skin areas, no differences in incidence were observed between resettlers and the general German population, suggesting that there are no other risk factors for resettlers. The observed convergence of disease incidence over time could be partially explained by medical underuse in the country of origin and direct access to medical care in Germany after immigration. However, a survey showed that despite direct access to the German health care system, resettlers tend not to participate in offered preventive and screening examinations [35, 36]. Studies have shown that cancer and cardiovascular disease rates of migrants adapt to rates of the host country [37–39].

The strengths of our study include the representative resettler cohort with an observation period of 18 years and complete follow-up for more than 95%, as well as the high quality of the Saarland Cancer Registry data [40–42]. However, we want to highlight that cancer registry data reflect the number of diagnosed cases and not necessarily the true incidence in a population. Therefore, NMSC incidence might be underestimated in particular before the introduction of skin cancer screening [43].

In contrast, the classification according to UV exposure represents a limitation, as this could only be approximated using tumour location. Concentrated in the first years of observation, in approximately 10% of the diagnoses, the tumour location was indicated as unknown (ICD-9: 173.9). A further classification into strongly, moderately, weakly and or UV unexposed locations was also waived with in this study due to the limited number of observations. The lack of individual UV exposure also hinders the observation of differences due to gender and fashion. However, viewed over a lifetime, it is predominantly the head and neck area that is exposed to UV radiation [6, 16]. Lacking individual information such as details on the region of origin, occupation and leisure time activity should also be noted.

Further, our analyses do not take in account the length of stay in Germany. However, immigration pattern of resettler show that the vast majority immigrated between 1990 and 1995 leading to high correlation of calendar year and length of stay.

Finally, we did not calculate ASR for resettler cohort in addition to the indirect SIR due to the relatively low number of cases.

## Conclusion

In summary, we were able to show that resettlers from the former Soviet Union carried an increased risk of developing non-melanoma skin cancer. Based on the distribution of the tumour sites, it can be assumed that a stronger UV exposure in the countries of origin contributed significantly to the increased risk. The combination of increased risk and possible underuse due to non-participation in screening and early detection programs, may result in an increased risk profile. Future studies should include the period after the introduction of skin cancer screening to show whether specific services are needed for the group of resettlers.

## Data Availability

Data of this study are not open access, but we encourage other researchers to contact us and apply for data access based on collaborating projects. Please, contact Volker Winkler (volker.winkler@uni-heidelberg.de) or Evgenia Markeeva-Ilisevic (E.Markeeva@stud.uni-heidelberg.de) to apply for possible collaborations.

## Abbreviations

ASR: Age-standardized incidence rates
BCC: Basal cell carcinoma
ICD-O2: International Classification of Disease for Oncology 2^nd^ Revision
NMSC: Non-melanoma skin cancer
SCC: Squamous cell carcinoma
SIR: Standardized incidence ratios
UV: Ultraviolet
